# RNA-Seq Reveals Activation of Both Common and Cytokine-Specific Pathways following Neutrophil Priming

**DOI:** 10.1371/journal.pone.0058598

**Published:** 2013-03-06

**Authors:** Helen L. Wright, Huw B. Thomas, Robert J. Moots, Steven W. Edwards

**Affiliations:** 1 Institute of Integrative Biology, University of Liverpool, Liverpool, Merseyside, United Kingdom; 2 Institute of Ageing and Chronic Disease, University Hospital Aintree, University of Liverpool, Liverpool, Merseyside, United Kingdom; University of California Riverside, United States of America

## Abstract

Neutrophils are central to the pathology of inflammatory diseases, where they can damage host tissue through release of reactive oxygen metabolites and proteases, and drive inflammation via secretion of cytokines and chemokines. Many cytokines, such as those generated during inflammation, can induce a similar “primed” phenotype in neutrophils, but it is unknown if different cytokines utilise common or cytokine-specific pathways to induce these functional changes. Here, we describe the transcriptomic changes induced in control human neutrophils during priming *in vitro* with pro-inflammatory cytokines (TNF-α and GM-CSF) using RNA-seq. Priming led to the rapid expression of a common set of transcripts for cytokines, chemokines and cell surface receptors (CXCL1, CXCL2, IL1A, IL1B, IL1RA, ICAM1). However, 580 genes were differentially regulated by TNF-α and GM-CSF treatment, and of these 58 were directly implicated in the control of apoptosis. While these two cytokines both delayed apoptosis, they induced changes in expression of different pro- and anti-apoptotic genes. Bioinformatics analysis predicted that these genes were regulated via differential activation of transcription factors by TNF-α and GM-CSF and these predictions were confirmed using functional assays: inhibition of NF-κB signalling abrogated the protective effect of TNF-α (but not that of GM-CSF) on neutrophil apoptosis, whereas inhibition of JAK/STAT signalling abrogated the anti-apoptotic effect of GM-CSF, but not that of TNF-α (p<0.05). These data provide the first characterisation of the human neutrophil transcriptome following GM-CSF and TNF-α priming, and demonstrate the utility of this approach to define functional changes in neutrophils following cytokine exposure. This may provide an important, new approach to define the molecular properties of neutrophils after *in vivo* activation during inflammation.

## Introduction

Neutrophils are professional phagocytes that play a critical role in host defence through the clearance of bacterial pathogens. Despite being the most abundant leukocyte found in human peripheral blood, the neutrophil has long been regarded as a primary response cell with little ability to influence the intracellular signalling events that are orchestrated by other immune cells during inflammation. However, in recent years there has been a greater appreciation of the direct role of inflammatory neutrophils in diseases such as rheumatoid arthritis (RA), chronic obstructive pulmonary disease (COPD) and juvenile systemic lupus erythematosus (JSLE) [1], [2], [3]. Neutrophils are activated by inflammatory stimuli to secrete reactive oxygen species (ROS) and proteases, which can damage host tissue if released inappropriately [4]. In addition, neutrophils drive inflammation via the secretion of inflammatory molecules such as cytokines, chemokines and leukotrienes [5]. Neutrophil secretory products such as myeloperoxidase, elastase, gelatinase, interleukin-8 and leukotriene-B_4_ are found in high concentrations at sites of inflammation, such as RA synovial fluid [6], [7], [8], [9] and the COPD lung, [10] and neutrophils have been shown to be critical to the initiation and progression of inflammatory arthritis in animal models of disease [11]. Many drugs now used to treat inflammatory diseases can decrease neutrophil migration and degranulation [12], [13], and we recently showed that neutrophil phenotype is modulated during treatment of RA with anti-TNF therapy, in line with improvements in disease activity [14].

Neutrophil function *in vivo* is regulated or “primed” by cytokines and chemokines generated during an inflammatory response. Priming induces a number of rapid (<1 h), functional changes, such as partial assembly of the NADPH oxidase, mobilisation of intracellular granules containing pre-formed receptors to the plasma membrane, and changes in the expression level and/or affinity of adhesion molecules such as integrins. A variety of agents, such as TNF-α, IL-1β, GM-CSF and IL-8, can induce neutrophil priming *in vitro* and these all induce a similar, primed phenotype resulting from these short-term molecular re-arrangements. For this reason, these agents are often used interchangeably to induce “neutrophil priming”, on the assumption that they induce these molecular changes via common mechanisms. This is unlikely to be the case. Also, it is known that these cytokines can regulate gene expression, but few studies have examined global gene expression patterns activated in primed neutrophils, and even fewer have directly compared patterns of gene expression triggered by different cytokines. Furthermore, the functional consequences on neutrophil function of this activated gene expression are largely unknown. We hypothesised that different cytokines may induce similar phenotypic changes in the neutrophil, but induce these changes via activation of different signalling pathways leading to differential gene activation. In view of the development of anti-cytokine drugs and inhibitors of signalling pathways for the treatment of inflammatory disease, it is extremely important to define the effects of specific cytokines on neutrophil gene expression, in order to predict the consequences of therapeutic blockade on the function of these cells and to select the appropriate drug.

In this study we used whole transcriptome sequencing to measure the effect of two commonly (and interchangeably) used priming agents, TNF-α and GM-CSF, on the global gene expression profile of healthy neutrophils. The aims of this work were three-fold. First, we wanted to characterise the changes in gene expression stimulated during *in vitro* “priming” of neutrophils. For this purpose, we treated neutrophils for 1 h with TNF-α and GM-CSF, as both of these cytokines are elevated in inflammatory diseases such as RA [9], and have previously been shown to prime neutrophils *in vitro* [15], [16], [17], . We measured the changes in gene expression using whole transcriptome sequencing (RNA-seq) which provides accurate quantification of gene expression. Secondly, we wanted to use these transcriptome data to identify which signalling pathways and transcription factors were activated by TNF-α and GM-CSF during rapid priming of neutrophils. Finally we wanted to validate any bioinformatics predictions using functionally relevant assays.

## Methods

### Ethics Statement

This study was approved by the University of Liverpool CORE (Committee on Research Ethics) and all participants gave written, informed consent.

### Isolation of Neutrophils

Blood was collected in lithium-heparin vacutainers from healthy controls. Neutrophils were isolated using Polymorphprep (Axis Shield), and contaminating erythrocytes were removed by hypotonic lysis. Freshly isolated neutrophils were incubated at 5×10^6^ cells/mL in RPMI 1640 media plus HEPES (Gibco) at 37°C with gentle agitation for 1 h in the absence (control) or presence of TNF-α (10 ng/mL, Calbiochem) or GM-CSF (5 ng/mL, Roche).

### Isolation of RNA

RNA was isolated from 3×10^7^ neutrophils using TRIzol-chloroform (Invitrogen) precipitation as per the manufacturer’s protocol. The RNA precipitate was cleaned up using an RNeasy mini kit (Qiagen), which included a DNA digestion step. Total RNA concentration and integrity was assessed using the Agilent 2100 Bioanalyser RNA Nano chip. RNA integrity (RIN) was routinely found to be ≥8.0.

### Library Generation and Sequencing

Total RNA was enriched for mRNA using ribosomal depletion (SOLiD) or poly-A selection (Illumina). Standard Illumina and SOLiD protocols were used to generate 50 bp single-end read libraries. Briefly, mRNA was fragmented, reverse transcribed, adapted with sequencing primers and sample barcodes, size selected and PCR enriched. The three barcoded libraries were sequenced together on half an ABI SOLiD v4.0 slide, or one lane of an Illumina HiSeq 2000 Analyser.

### Read Mapping and Gene Annotation

Reads were mapped to the human genome (hg19) using TopHat [21], [22] and Bowtie [23], and annotated using Cufflinks [24]. A minimum RPKM expression threshold of ≥0.3 was applied to the data in order to minimise the risk of including false positives against discarding true positives from the dataset [25], [26]. Statistical analysis was carried out using Cuffdiff [24], and visualised using MeV [27]. Further details, including mapping parameters are described in Methods S1 and the number of reads mapped in each library are detailed in Table S1. The data reported in this manuscript have been deposited in the NCBI’s Gene Expression Omnibus (GEO) and are accessible through GEO Series accession number GSE40548 (http://www.ncbi.nlm.nih.gov/geo/query/acc.cgi?acc=GSE40548).

### Bioinformatics

Bioinformatics analysis was carried out using DAVID [28] and IPA (Ingenuity® Systems, www.ingenuity.com). Hierarchical cluster analysis was carried out using MeV [27] using euclidean clustering and average linkage. Further details are provided in Methods S1.

### Real-time PCR

cDNA was synthesised using the Superscript III First Strand cDNA Synthesis kit (Invitrogen) using equal concentrations of RNA across samples, as per the manufacturer’s instructions. Real-time PCR analysis was carried out using the QuantiTect SYBR Green PCR kit (Qiagen) as per the manufacturer’s instructions. Analysis was carried out on a Roche 480 LightCycler in a 96-well plate using a 20 µL reaction volume. Target gene expression was quantified against a panel of housekeeping genes (GAPDH, B2M, ACTB, PPIA) [29]. Primer sequences can be found in Table S2.

### Measurement of the Respiratory Burst

Neutrophils (5×10^6^/mL) were incubated with TNF-α (10 ng/mL) or GM-CSF (5 ng/mL) for up to 1 h. Cells (10^6^) were re-suspended in HBSS (Gibco) containing luminol (10 µM, Sigma) and the respiratory burst was stimulated with fMLP (1 µM, Sigma). Luminescence was measured using an LKB 1251 luminometer at 37°C.

### Antibody Staining

Antibody staining was carried out on freshly isolated neutrophils and on control neutrophils that had been incubated for 1 h with or without TNF-α (10 ng/mL), or GM-CSF (5 ng/mL). Neutrophils (5×10^4^) were resuspended in PBS (plus 0.2% BSA). Antibody binding was carried out at 4°C in the dark for 30 min with conjugated antibodies added as follows: CD11b-FITC (Miltenyi Biotec), CD18-FITC (R&D systems), L-selectin-FITC (R&D systems), CD16 (BD Biosciences), CD32 (BD Biosciences), FITC-isotype controls (Santa Cruz). Cells were fixed with 2% paraformaldehyde and fluorescence was measured on a Guava EasyCyte flow cytometer. 5,000 events per sample were analysed.

### Measurement of Apoptosis

Neutrophils (10^6^/mL) were incubated with the signalling inhibitors, wedelolactone (50 µM) and JAK inhibitor-1 (10 µM), (both Calbiochem) for 1 h prior to the addition of TNF-α (10 ng/mL) or GM-CSF (5 ng/mL), and incubated at 37°C with 5% CO_2_ for 18 h. Neutrophils (2.5×10^4^) were then stained with Annexin V-FITC (Invitrogen) for 15 min. Propidium-iodide (1 µg/mL, Sigma) was added prior to analysis on a Guava EasyCyte flow cytometer. 5,000 events were analysed per sample.

### Western Blotting of Phosphorylated Proteins

Neutrophils (5×10^6^/mL) were incubated with signalling inhibitors (wedelolactone, 50 µM; JAK inhibitor-1, 10 µM) for 1 h prior to the addition of TNF-α (10 ng/mL) or GM-CSF (5 ng/mL) for 15 min. Neutrophils were centrifuged at 1000g for 3 min, and rapidly lysed in boiling Laemmli buffer containing phosphatase inhibitor cocktail II (Calbiochem). Protein samples (10^5^ cells) were separated by SDS-PAGE using a 10% gel and transferred onto PVDF membrane (Millipore). Primary antibodies were: phosphorylated NF-κB (p65), IκB-α, phosphorylated STAT-3, (all 1∶1000, Cell Signaling), and GAPDH (1∶10,000, Abcam). Secondary antibodies were anti-rabbit IgG (GE Healthcare) and anti-mouse IgG (Sigma) HRP-linked antibodies (1∶10,000). Bound antibodies were detected using the ECL system (Millipore) on carefully exposed film (Amersham) to avoid saturation.

## Results

### Neutrophil Priming by TNF-α and GM-CSF

**Figure 1:**
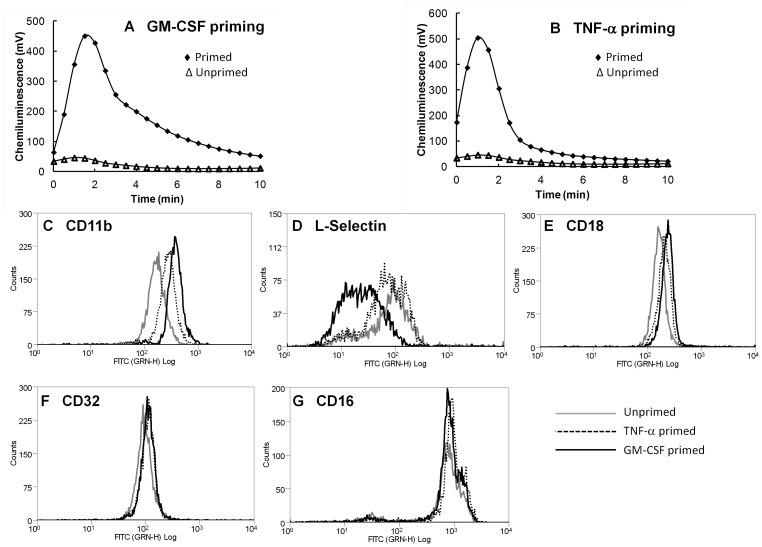
Effect of GM-CSF and TNF-α on neutrophil priming. (A,B) Neutrophils were primed with (A) GM-CSF (5 ng/mL) or (B) TNF-α (10 ng/mL), and the respiratory burst was stimulated by fMLP (10 µM). A rapid respiratory burst was observed in primed cells (♦) but not in unprimed cells (Δ). (C–G) Flow cytometry analysis of adhesion molecule expression following priming with GM-CSF (black line) or TNF-α (dashed line) compared to unprimed neutrophils (grey line). (C) CD11b and (E) CD18 expression was up-regulated following priming with both GM-CSF and TNF-α but showed a greater level of up-regulation after GM-CSF priming. (D) L-selectin showed significant shedding following priming with GM-CSF, but only moderate shedding after TNF-α priming. (F) FcγRIIA (CD32) expression did not change following priming with either cytokine, and (G) FcγRIIIB expression was maintained by priming with either cytokine compared to the level of expression in untreated neutrophils from which the receptor was shed during 1 h incubation.

In order to compare the functional changes induced during neutrophil priming by TNF-α and GM-CSF, we firstly measured the respiratory burst generated by unprimed and primed neutrophils in response to the bacterial peptide fMLP. Both TNF-α and GM-CSF primed neutrophils generated a rapid respiratory burst in response to fMLP, which peaked at around 2 min exposure to the peptide (Figure 1A,B). No respiratory burst was generated in unprimed neutrophils in line with previously published results [30]. We next measured the ability of TNF-α and GM-CSF to up-regulate expression of the α_2_β_M_-integrin (Mac-1) subunits CD11b and CD18. Priming with GM-CSF or TNF-α for 1 h up-regulated expression of both CD11b (Figure 1C) and CD18 (Figure 1E), but to a greater extent in GM-CSF primed neutrophils. The adhesion molecule, L-selectin was shed to a greater extent following 1 h priming with GM-CSF, while TNF-α priming induced only moderate shedding of this molecule (Figure 1D). The FcγRIIA (CD32) receptor was not up-regulated by priming with either cytokine (Figure 1F), and both TNF-α and GM-CSF maintained expression of FcγRIIIB (CD16, Figure 1G) which is normally shed during the culture of unstimulated neutrophils, in line with increased rates of apoptosis [31]. Taken together these results indicate that these two cytokines induce subtle differences in neutrophil phenotype during the priming response.

### Sequencing of the Neutrophil Transcriptome

**Figure 2:**
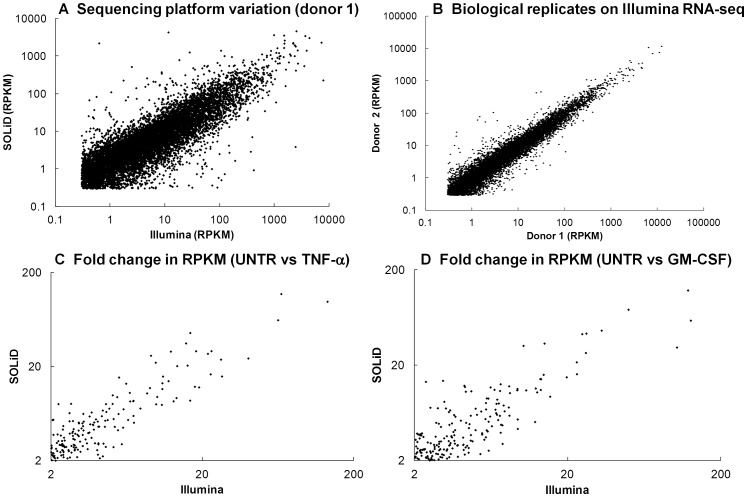
Comparison of sequencing platform variation and biological variation. (A) RPKM values (≥0.3) for untreated neutrophils from the same donor sequenced on the SOLiD v4.0 and Illumina HiSeq 2000 platforms (Rs = 0.784, Pearson Correlation). (B) RPKM values (≥0.3) for untreated neutrophils from two different biological donors sequenced on the Illumina HiSeq 2000 platform (Rs = 0.947, Pearson Correlation). (C,D) Correlation between the fold change in RPKM value for genes up-regulated by (C) TNF-α (Rs = 0.885) and (D) GM-CSF (Rs = 0.831) measured in neutrophils from the same donor on the SOLiD v4.0 and Illumina HiSeq 2000 platforms.

In order to investigate the different molecular changes induced during priming of neutrophils by TNF-α and GM-CSF, we carried out whole transcriptome analysis on mRNA isolated from 1 h primed and unprimed neutrophils. The transcriptomes from cytokine treated (TNF-α or GM-CSF) and untreated human neutrophils were sequenced on both the Illumina HiSeq2000 and ABI SOLiD v4.0 platforms. Neutrophil RNA from one donor was sequenced on both platforms to compare inter-platform variability, and neutrophil RNA from two different donors was sequenced on the Illumina platform to compare donor-donor variation. Gene expression (RPKM) [32] measured across the two platforms (SOLiD and Illumina) showed significant correlation (Figure 2A, p<2.2E–16, Rs = 0.784, Pearson correlation). The Pearson correlation for the two biological replicates on the Illumina platform was 0.947 (Figure 2B), and this is broadly in line with transcriptomic studies carried out on other cell types [26], [33], [34]. The lower Pearson correlation for the between-platform comparison may be explained by a number of factors, such as differing mRNA enrichment protocols and mapping strategies, which we detail in Methods S1. Despite a lower between-platform correlation of absolute gene expression (RPKM) values, we found a high level of correlation in the fold change of gene expression induced by TNF-α (Figure 2C) and GM-CSF (Figure 2D) measured on each platform, of 0.886 and 0.831 respectively (Pearson correlation). This suggests that whilst absolute RPKM values may not correlate well between platforms, the biological information, i.e. the relative change in gene expression, shows a good correlation between independent sequencing platforms.

**Figure 3:**
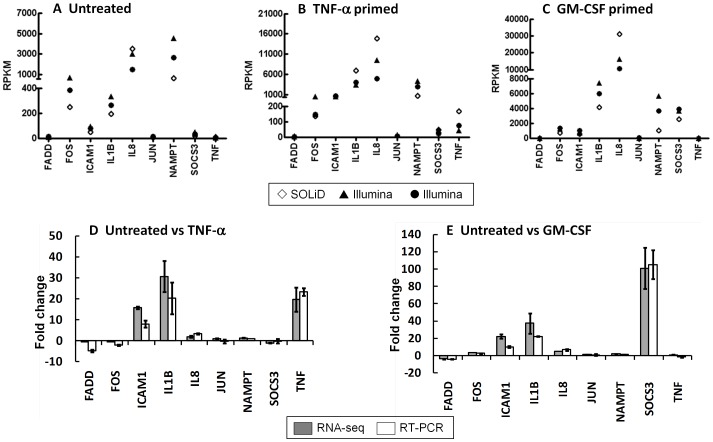
Validation of expression values of a selection of genes measured by RNA-seq and real-time PCR. (A–C) Expression levels of a selection of genes with a range of RPKM values across the two NGS platforms (⋄ SOLiD, n = 1, •▴ Illumina, n = 2) in (A) untreated, (B) TNF-α-treated and (C) GM-CSF-treated neutrophils. Symbols overlap at some datapoints due to highly similar RPKM values. (D,E) Fold change in expression of genes in (D) TNF-α and (E) GM-CSF-treated neutrophils compared to unstimulated, measured by real-time PCR (grey bar, n = 3) and RNA-seq (open bar, n = 3).

In order to validate the sequencing, we decided to investigate the expression of a set of genes with a range of RPKM values to determine (a) the biological variation in expression of these genes, and (b) whether genes with low RPKM values could be detected by PCR. We selected genes with high (>3000) RPKM values (IL8, NAMPT, SOCS3), mid-range (50–3000) RPKM values (FOS, ICAM1, IL1B) and low (<50) RPKM values (FADD, JUN, TNF). The RPKM values of these genes in each sample (unstimulated, TNF-α-primed, GM-CSF-primed) from the three sequencing datasets (SOLiD donor 1, Illumina donor 1 and Illumina donor 2) are shown in Figure 3 (A–C). We found that, in the main, RPKM values showed less donor-donor variation than platform variation (e.g. IL1B, NAMPT and SOCS3, Fig. 3A–C). Where there was a wider variation of RPKM values between donors, we found that the fold changes in RPKM values after cytokine treatment were highly similar. For example, whilst the transcript for IL8 in untreated neutrophils had an RPKM value of 3544 on the SOLiD platform and 1497 on the Illumina platform for the same donor, the fold-change in RPKM values for IL8 between untreated and GM-CSF primed neutrophils were 4-fold and 3-fold, measured by SOLiD and Illumina, respectively. We next carried out real-time PCR analysis of these genes using neutrophil RNA from three healthy individuals who were not the donors for the neutrophils which were sequenced (Figure 3D–E). We were able to detect all genes by PCR (C_T_ value <30). The transcript for TNF in untreated neutrophils had the lowest RPKM value of the genes we investigated and this corresponded to a C_T_ value of 26.4±1.2 The fold changes in gene expression between untreated neutrophils and cytokine-treated neutrophils measured by real-time PCR showed high comparability with the fold changes in RPKM values for the same genes quantified in the RNA-seq datasets (Figure 3D,E).

### Differently Expressed Genes in Cytokine Treated Neutrophils

**Figure 4:**
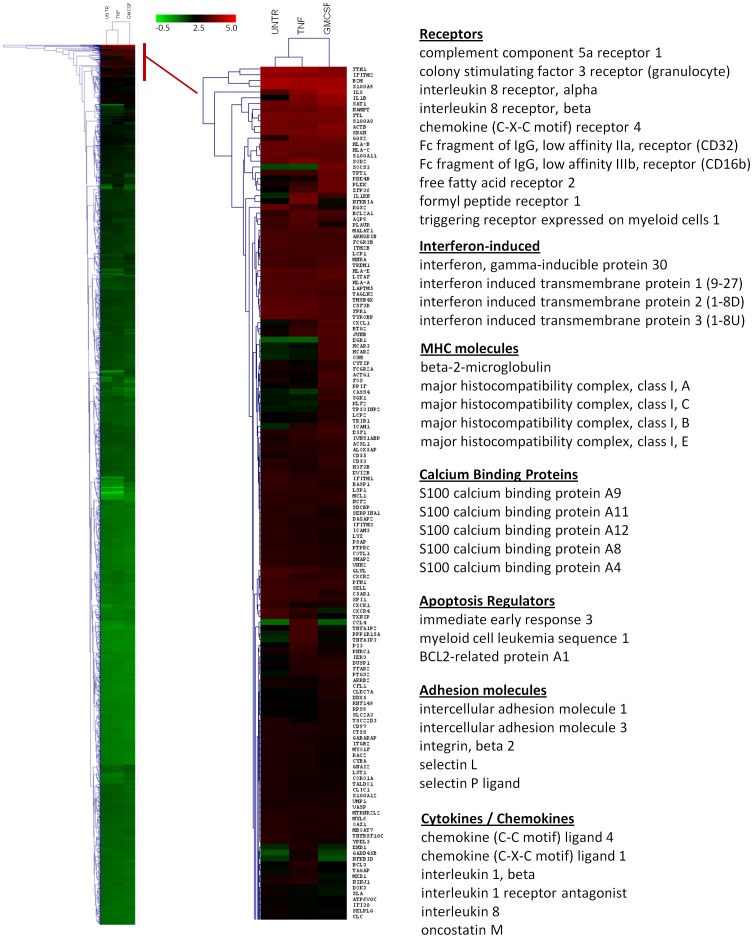
Hierarchical cluster analysis of genes expressed (RPKM ≥10) in untreated and cytokine treated neutrophils. RPKM values are represented on a log_10_ scale, where green is low expression and red is high expression. An expanded heat map of highly expressed genes (red bar) is also shown. These highly-expressed transcripts include genes that can be categorised as cytokines/chemokines, cell-surface receptors, interferon-induced genes, Major Histocompatibility Complex (MHC) proteins, calcium binding proteins, apoptosis regulators and adhesion molecules.

Analysis of RNA isolated from unstimulated neutrophils revealed expression (RPKM ≥0.3) of 11,242 known genes, which is in broad agreement with previously published data obtained by micro-array hybridisation experiments [35], [36]. Hierarchical cluster analysis of all genes with an RPKM ≥10 in at least one of the three datasets (untreated, TNF-α, GM-CSF) is shown in Figure 4. An expanded heat map of the most highly expressed genes is also shown in Figure 4. These highly-expressed transcripts include genes that can be categorised as cytokines/chemokines, cell-surface receptors, interferon-induced genes, Major Histocompatibility Complex (MHC) proteins, calcium-binding proteins, apoptosis regulators and adhesion molecules.

Statistical analysis of differentially expressed (DE) genes was carried out using the Cufflinks tool Cuffdiff, applying a 5% false discovery rate (FDR). Of the genes which were upregulated (≥1.5 fold) by TNF-α, 251 genes reached statistical significance (FDR <0.05). This compares to 505 genes in GM-CSF primed neutrophils. Likewise, cytokine treatment resulted in down-regulation of a number of genes: in TNF-α-treated neutrophils, 345 genes were down-regulated at least 1.5 fold compared to untreated controls, and GM-CSF treatment led to down-regulation of 1338 genes.

We found that 40 significantly DE genes were up-regulated by at least 10-fold in TNF-α and/or GM-CSF treated neutrophils (Table 1). Interestingly, genes for chemokines were differently expressed with the two cytokine treatments: CCL3 and CCL4 were only up-regulated by TNF-α treatment, CXCL1 was up-regulated around 3-fold greater by GM-CSF compared to TNF-α, and CXCL2 was up-regulated over 6-fold greater by TNF-α compared to GM-CSF. The cytokines IL-1A and IL-1B were up-regulated by both stimuli, whereas oncostatin M (OSM) was only up-regulated by GM-CSF. Expression of the TNF-α gene (TNF) was only stimulated by TNF-α and not GM-CSF.

**Table 1:** Genes up-regulated at least 10-fold in TNF-α and/or GM-CSF treated neutrophils compared to untreated neutrophils.

Gene	TNF-α	GM-CSF
**CCL3**	41.5	NS
**CCL4**	99.6	NS
**CD69**	NS	57.5
**CISH**	NS	102.1
**CXCL1**	3.6	10.4
**CXCL2**	29.0	4.7
**DUSP2**	12.1	NS
**EDN1**	NS	16.5
**EGR1**	NS	57.9
**EGR2**	NS	21.5
**GADD45B**	15.8	NS
**GPR84**	74.9	NS
**HBEGF**	NS	33.2
**HCAR2**	NS	12.1
**HCAR3**	NS	12.2
**HRH4**	NS	32.3
**ICAM1**	7.7	10.6
**IL1A**	67.0	35.3
**IL1B**	13.8	22.8
**IL1RN**	31.4	12.4
**KCNJ2**	16.6	NS
**MFSD2A**	11.9	−1.6
**NFKBIA**	11.9	NS
**NFKBIE**	15.8	NS
**OLR1**	3.2	NS
**OSM**	NS	15.0
**PDE4B**	NS	11.8
**PLAU**	13.9	5.8
**PNPLA1**	10.2	−2.1
**PPP1R15A**	10.2	3.6
**RHOH**	NS	26.3
**SLC35B2**	10.4	NS
**SOCS3**	NS	90.2
**TARP**	NS	13.5
**TIFA**	17.7	6.7
**TNF**	25.8	NS
**TNFAIP3**	16.1	2.7
**TNFAIP6**	10.6	NS
**TRAF1**	11.6	NS
**ZFP36**	4.7	11.4

Table shows fold change in gene expression (RPKM) compared to level expressed in untreated neutrophils. Change in gene expression is significant with a 5% FDR (NS = not significant).

In order to characterise this sub-set of genes showing DE during neutrophil priming with TNF-α or GM-CSF, we carried out Gene Ontology (GO) analysis using DAVID [28]. GO analysis is a useful bioinformatics tool to categorise and group large gene sets based on a known functional association, as defined by the Gene Ontology Consortium [37]. GO terms are hierarchical and describe biological processes and metabolic functions that are uniform across species. This is explained in depth in the GO Consortium publication [37], but for example, a “high level” or broadly descriptive GO term would be “cell growth and maintenance” or “signal transduction”, whereas a more specific “low level” GO term would be “pyrimidine metabolism” or “cAMP biosynthesis”. We found that the genes which were significantly DE during priming of neutrophils with TNF-α or GM-CSF led to enrichment of both common and cytokine-specific ontologies, as summarised in Table 2. High level, or broadly descriptive GO categories such as “immune response” and “defense response” were represented in both TNF-α and GM-CSF primed neutrophils. More specific, lower level GO categories were enriched in neutrophils primed by only one of the cytokines, such as “chemotaxis” and “regulation of I-kappaB kinase/NF-kappaB cascade” in TNF-α primed neutrophils.

**Table 2:** Gene ontology analysis of genes with differential expression during priming by TNF-α or GM-CSF.

GO Term	GO Category	GM-CSF	TNF-α
GO:0006954	inflammatory response	*	*
GO:0009611	response to wounding	*	*
GO:0006955	immune response	*	*
GO:0042981	regulation of apoptosis	*	*
GO:0006952	defense response		*
GO:0006935	chemotaxis		*
GO:0043122	regulation of I-kappaB kinase/NF-kappaB cascade		*
GO:0007243	protein kinase cascade		*
GO:0031328	positive regulation of cellular biosynthetic process	*	
GO:0010557	positive regulation of macromolecule biosynthetic process	*	
GO:0010628	positive regulation of gene expression	*	
GO:0045321	leukocyte activation	*	
GO:0010604	positive regulation of macromolecule metabolic process	*	
GO:0001775	cell activation	*	
GO:0045766	positive regulation of angiogenesis	*	
GO:0051789	response to protein stimulus	*	
GO:0008285	negative regulation of cell proliferation	*	
GO:0051174	regulation of phosphorus metabolic process	*	
GO:0019220	regulation of phosphate metabolic process	*	
GO:0032570	response to progesterone stimulus	*	
GO:0042325	regulation of phosphorylation	*	
GO:0045429	positive regulation of nitric oxide biosynthetic process	*	
GO:0006350	transcription	*	
GO:0045893	positive regulation of transcription, DNA-dependent	*	
GO:0051254	positive regulation of RNA metabolic process	*	
GO:0045859	regulation of protein kinase activity	*	

GO analysis was carried out using DAVID revealing common and cytokine-specific changes induced during 1 h priming (*represents an FDR adjusted q-value ≤0.05).

**Figure 5:**
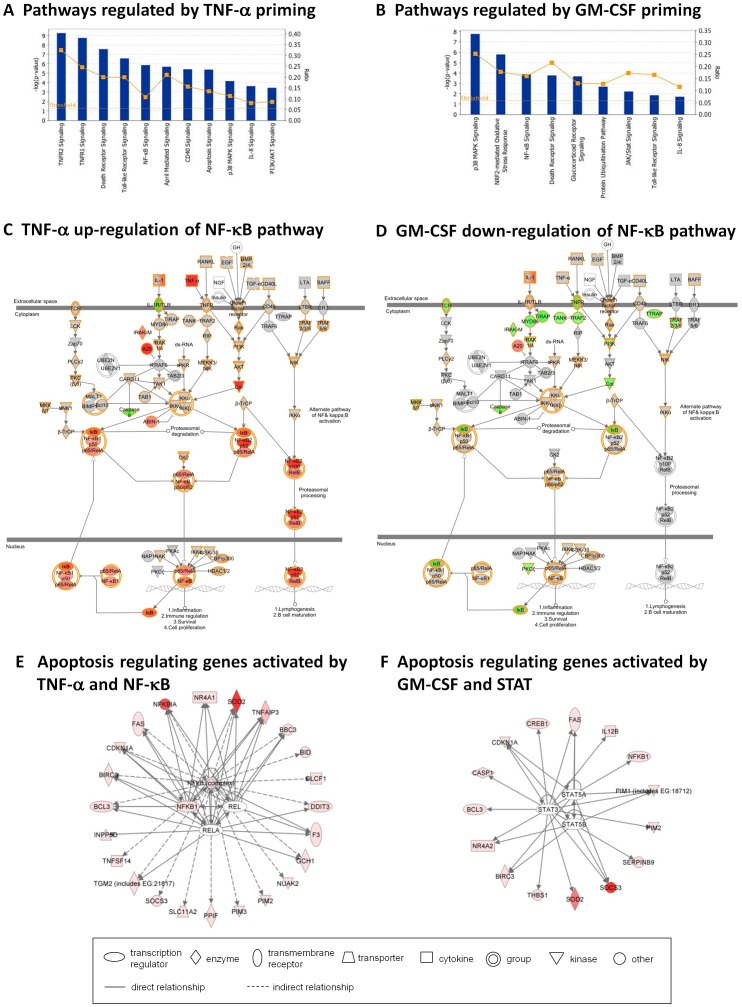
Functional analysis of signalling pathway expression in TNF-α and GM-CSF treated neutrophils. (A, B) Bar graphs showing the pathways with the most significant changes in regulation in cytokine treated neutrophils compared to untreated neutrophils. The bars represent the p-value of the probability that the association between the genes in the dataset (A, TNF-α, B, GM-CSF) and the canonical pathway, is due to chance alone. The orange line represents the ratio of the number of genes in the dataset compared to the number of genes in the canonical pathway. (C, D) The NF-κB pathway was identified in both TNF-α (C) and GM-CSF (D) datasets as being differently regulated compared to untreated neutrophils. Up-regulated genes are shown in red, down-regulated genes are shown in green, and genes with no change in expression level are shown in grey. (E, F) IPA analysis of 58 apoptosis regulating genes with significant DE expression between TNF-α and GM-CSF treated neutrophils. (E) NF-κB transcription factor activation was predicted in TNF-α-treated neutrophils (p = 9.04E–11), whereas STAT activation (B) was predicted in GM-CSF-treated neutrophils (p = 2.26E–05). The RPKM value of individual genes is represented by increasing intensity of red.

Whilst GO analysis is a useful tool to describe the cellular processes that are enriched by a set of genes, it is unable to predict activation of specific signalling pathways. Therefore to supplement our GO analysis, we carried out functional analysis of DE genes using Ingenuity (IPA). This revealed significant changes in the regulation of intracellular signalling pathways by TNF-α, including death-receptor signalling, NF-κB signalling, APRIL signalling, and apoptosis (Figure 5A). In contrast, GM-CSF treated neutrophils showed significant changes in regulation of signalling pathways such as p38 MAPK signalling and protein ubiquitination (Figure 5B). Our analysis identified signalling pathways whose regulation is changed following treatment, but does not distinguish whether those pathways are up- or down-regulated. An example of this is shown in Figure 5 C,D. The NF-κB pathway was identified as being significantly differentially regulated by both TNF-α and GM-CSF compared to the level of expression in untreated neutrophils. By overlaying the fold change in expression of each gene onto the canonical pathway it is possible to visualise which parts of the pathway are up-regulated (red), down-regulated (green) or show no change in expression (grey) within each dataset (5C, TNF-α; 5D, GM-CSF) compared to untreated neutrophils. Differential regulation of NF-κB target genes within the TNF-α and GM-CSF treated neutrophils will be discussed in more detail later.

**Figure 6:**
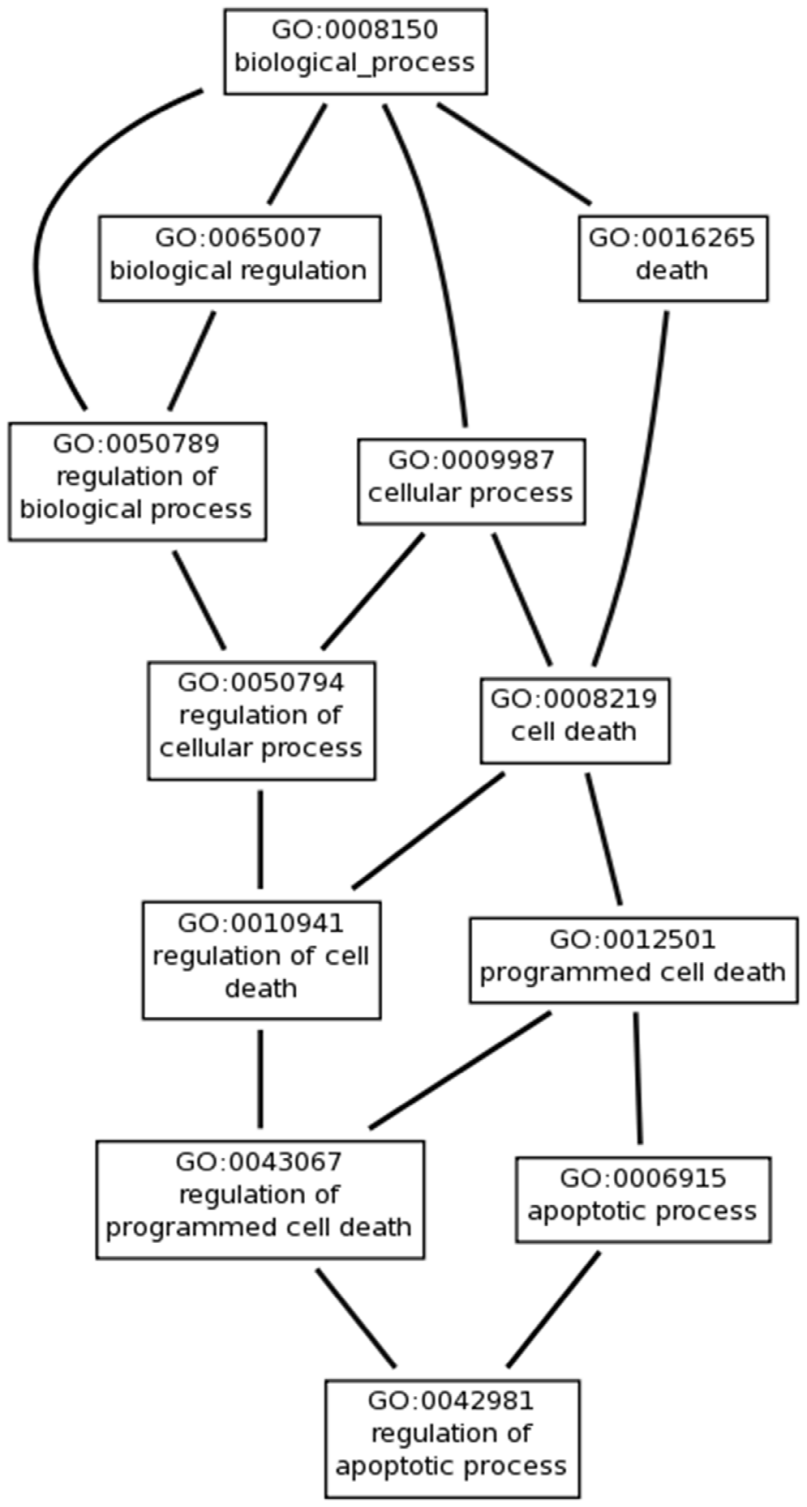
Gene Ontology tree of apoptosis-related GO categories. These GO categories were significantly enriched by genes which were differentially regulated in TNF-α and GM-CSF treated neutrophils.

Cuffdiff analysis also identified 580 genes that were significantly DE between TNF-α and GM-CSF treated neutrophils. GO analysis of these genes was carried out and those categories that were significantly enriched (FDR <5%) are summarised in Table S3. The most represented GO category was “Regulation of apoptosis” which contained 58 genes from this dataset. Interestingly, of the 45 significantly-enriched GO categories, 11 related to the regulation of cell death, and the hierarchy of these GO categories is shown in Figure 6. A similar result was obtained by analysing the 580 DE genes using IPA, which identified “Apoptosis” as the cellular function with greatest significance of differential regulation between the two treatments (p = 6.78E–23). The expression values of the 58 apoptosis-related genes with DE in TNF-α and GM-CSF treated neutrophils are shown in Table 3. In order to further investigate the differences in regulation of this subset of 58 apoptotic genes between TNF-α and GM-CSF stimulation, we used IPA to predict transcription factor activation in the two datasets. Thirty-seven genes were more highly expressed in TNF-α treated neutrophils, and of these, 23 were predicted to be regulated by the NF-κB transcription factor complex (p = 5.77E–21), Figure 5E. Conversely, 15 of the 21 genes that were more highly expressed in GM-CSF treated neutrophils, were predicted to be regulated by the STAT family of transcription factors (p = 2.73E–12), in particular STAT3 and STAT5, Figure 5F.

**Table 3:** The 58 apoptosis-related genes which had significantly different expression levels in TNF-α and GM-CSF treated neutrophils (FDR adjusted q-value ≤0.05).

Gene	TNF-α	GM-CSF
**ANXA1**	82.28	231.73
**APAF1**	24.50	20.50
**BBC3**	15.43	3.58
**BCL3**	764.80	323.05
**BID**	192.62	66.72
**BIRC3**	107.60	14.36
**CARD16**	114.51	68.22
**CARD6**	2.73	12.14
**CASP1**	87.50	65.68
**CDKN1A**	16.55	44.49
**CDKN2C**	1.09	0.37
**CHST11**	79.18	61.81
**CLCF1**	6.73	0.95
**CREB1**	13.50	8.22
**DDIT3**	109.21	240.01
**F3**	2.98	0.80
**FAS**	62.78	33.88
**GCH1**	31.89	12.90
**GHRL**	12.84	6.28
**HSPD1**	8.29	20.84
**ID3**	<0.3	2.01
**INPP5D**	88.53	68.32
**MAEA**	57.98	27.03
**NET1**	0.60	5.76
**NFKB1**	42.05	21.19
**NFKBIA**	3901.19	225.87
**NLRP3**	49.76	16.81
**NR4A1**	21.41	2.02
**NR4A2**	50.14	18.14
**NUAK2**	24.99	5.25
**PIM1**	11.26	98.05
**PIM2**	260.17	36.66
**PIM3**	246.79	74.97
**PLAGL2**	33.72	4.81
**PPIF**	488.80	1352.77
**PRNP**	4.24	17.15
**PROK2**	210.88	582.65
**PSEN1**	69.94	39.99
**RIPK2**	36.63	92.35
**RRM2B**	16.15	7.70
**SERPINB9**	37.09	13.36
**SLC11A2**	1.82	0.78
**SMPD2**	9.13	2.04
**SOCS2**	2.96	23.61
**SOCS3**	36.45	4060.71
**SOD2**	4619.77	2696.87
**SQSTM1**	280.27	114.89
**TGM2**	0.68	4.84
**THBS1**	52.91	13.16
**TICAM1**	47.41	9.98
**TNFAIP3**	1451.80	244.30
**TNFRSF10D**	1.09	12.54
**TNFSF14**	85.44	41.61
**TNFSF15**	0.77	3.59
**TNFSF8**	5.93	19.93
**TPT1**	1310.06	3025.26
**UTP11L**	1.39	5.02
**ZAK**	0.51	1.40

Table shows the gene expression (RPKM) value of each gene following priming for 1 h with TNF-α or GM-CSF.

### Regulation of Neutrophil Apoptosis by TNF-α and GM-CSF via Activation of Different Transcription Factors

**Figure 7:**
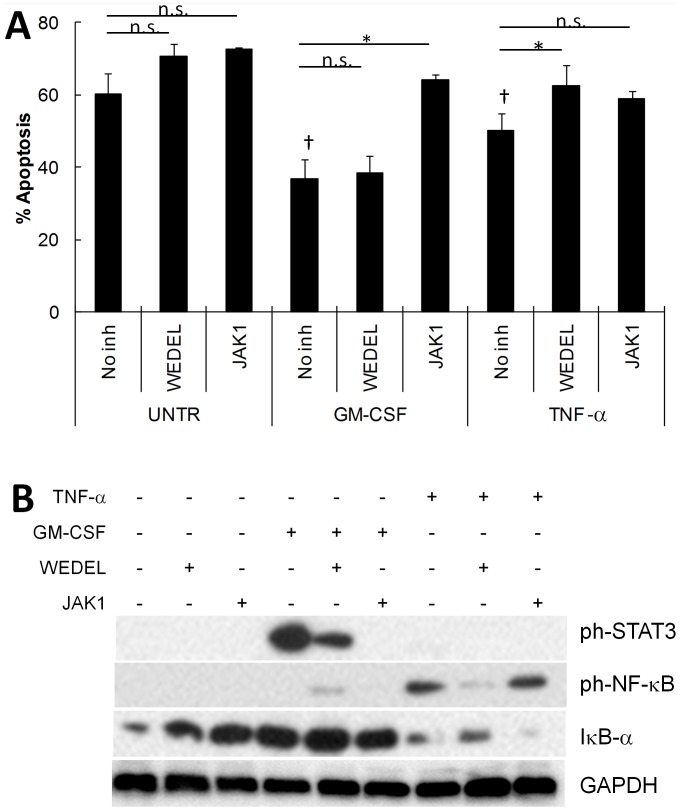
Delayed apoptosis in TNF-α and GM-CSF-treated neutrophils is regulated via different transcription factor activation. (A) TNF-α and GM-CSF delayed apoptosis in healthy neutrophils incubated for 18 h († p<0.05, Student’s t-test) compared to the constitutive rate of apoptosis seen in untreated (UNTR) neutrophils. Inhibition of STAT signalling with JAK inhibitor-1 (JAK1, 10 µM) abrogated the effect of GM-CSF on neutrophil apoptosis (*p<0.05) but did not affect TNF-delayed apoptosis. Inhibition of NF-κB with wedelolactone (WEDEL, 50 µM) abrogated the effect of TNF-α, but not GM-CSF, on neutrophil apoptosis (*p<0.05). (B) Western blot of NF-κB and STAT3 activation in TNF-α and GM-CSF treated neutrophils. TNF-α induced rapid phosphorylation of NF-κB (p65) and degradation of IκB-α, which was inhibited by wedelolactone. GM-CSF did not induce phosphorylation of NF-κB or degradation of IκB-α, but did induce STAT-3 phosphorylation which was inhibited by JAK inhibitor-1. TNF-α did not activate STAT-3 in neutrophils.

The above bioinformatics analyses indicated that while both TNF-α and GM-CSF result in expression of apoptosis-regulating genes, they do so via different signalling pathways leading to activation of different transcription factors. We therefore validated our bioinformatics analysis in functional assays: we incubated healthy neutrophils with TNF-α or GM-CSF in the presence of chemical inhibitors of NF-κB (wedelolactone, 50 µM) and JAK/STAT (JAK inhibitor-1, 10 µM). In line with previously published data [15], [16], TNF-α and GM-CSF delayed apoptosis of healthy neutrophils incubated *in vitro* for 18 h (Figure 7A). Inhibition of NF-κB using wedelolactone abrogated the anti-apoptotic effect of TNF-α (p<0.05, Student’s t-test), but had no effect on GM-CSF-delayed apoptosis. Conversely, inhibition of STAT using JAK inhibitor-1 abrogated GM-CSF-delayed apoptosis (p<0.05, Student’s t-test), and only partially attenuated TNF-α -delayed apoptosis (p>0.05).

Western blotting of protein lysates from neutrophils incubated with TNF-α or GM-CSF for 15 min in the presence of both inhibitors showed rapid activation of NF-κB and degradation of IκB-α by TNF-α, which was abrogated by wedelolactone but not by JAK inhibitor-1 treatment (Figure 7B). In contrast, GM-CSF was not able to activate NF-κB, but was able to rapidly phosphorylate STAT3, which was abrogated by JAK inhibitor-1.

## Discussion

In this study, we have investigated the changes in gene expression induced during *in vitro* cytokine priming of neutrophils, using a whole transcriptome sequencing approach (RNA-seq). We treated healthy neutrophils with two priming agents, TNF-α and GM-CSF, both of which are elevated during inflammation and in inflammatory disease [9]. Bioinformatics analyses have predicted differences in transcription factor activation by these two priming agents that initiate transcription of different sets of genes to regulate the functional responses observed in cytokine-primed neutrophils. We have validated these bioinformatics predictions by functional assays on cells incubated *in vitro*, and have shown that, whilst TNF-α and GM-CSF exert similar short-term (<1 h) functional effects on neutrophil priming, the post-priming phenotype of the neutrophil is mediated via the activation of distinct intracellular signalling pathways.

This study also provides the first study of global gene expression in healthy, unstimulated and cytokine-stimulated human neutrophils using RNA-seq technology. Whilst several published studies have used microarray technology to investigate changes in neutrophil gene expression induced by agonists such as GM-CSF [36] and LPS [38], [39], [40], our investigation provides the first analysis of neutrophils using RNA-seq, and our data have been made publically available via GEO. Both microarray and RNA-seq are established, robust technologies for the study of global gene expression, and have been shown to correlate well when the same biological samples have been analysed by both technologies [41], [42], [43], [44]. However, RNA-seq offers several advantages over microarray, as it allows estimation of absolute gene expression levels, and in particular, is not biased by signal saturation from high abundance genes. It also provides greater sensitivity for low abundance transcripts. Our study also provides the first direct comparison of the changes induced by two different cytokines on global gene expression in human neutrophils. Neutrophil studies have previously characterised the effect of single cytokines or agonists on global gene expression [36], [38], [40], and have then utilised real-time PCR to confirm changes in gene expression on a small sample of genes of interest with a larger number of agonists.

The functional effects of TNF-α and GM-CSF priming on healthy neutrophils *in vitro* have been described previously by ourselves and others [15], [16], [17], [18], [19], [20], and include delayed apoptosis, priming of the respiratory burst, altered expression of Fcγ receptors and increased expression/affinity of adhesion molecules. Priming involves both molecular re-arrangements to change the activity and/or sub-cellular localisation of pre-existing molecules, and also activation of gene expression. Examples of the former processes include rapid phosphorylation of the cytosolic phox components of the NADPH oxidase [17] and cytoskeletal rearrangements to mobilise intracellular granules and secretory vesicles containing membrane proteins from the cytoplasm to the plasma membrane [45]. Priming also results in activation of *de novo* biosynthesis, for example for the generation of cytokines and chemokines. Many of the functional effects of TNF-α and GM-CSF are similar, and yet our data show that these two cytokines activate different sets of transcription factors resulting in significant differential expression of several hundred genes.

The most highly up-regulated genes induced by priming healthy neutrophils with TNF-α included cytokines (IL1A, IL1B, IL1RN, TNF) and chemokines (CCL3, CCL4, CXCL2) which were all up-regulated by at least 10-fold. Interestingly, cytokine and chemokine production by primed neutrophils appears to be differentially regulated by TNF-α and GM-CSF. This is likely to play an important role in diseases where these cytokines are implicated, such as TNF-α in RA. The role of neutrophils in the production of cytokines and chemokines during inflammation is becoming more appreciated, and they are now considered critical regulators of both innate and adaptive immune responses [5], [46]. The role of chemokines in the pathogenesis of diseases, such as RA, is perhaps less well understood than that of cytokines, such as IL-1β and TNF-α (which are successfully targeted by biologic therapy in inflammatory disease [1]). However, numerous chemokines, including CCL3, CCL4, CXCL2 and IL-8, are elevated in both RA synovial tissue and synovial fluid, as well as in neutrophils isolated from RA joints [47], [48], [49], [50]. The success of anti-TNF therapy in treating patients with very active RA may therefore by explained, in part, by blockade of TNF-α-induced production of other mediators of inflammation, such as chemokines, by neutrophils and other immune cells. We also observed up-regulation of IL1A, IL8 and IL1B genes in GM-CSF stimulated healthy neutrophils. A study by Kobayashi et al. [36] investigated the effect of GM-CSF on neutrophil gene expression using microarrays. Whilst their study did not report genes associated with cytokine production, a number of genes up-regulated in our GM-CSF-treated neutrophil dataset (SOCS3, CD69, RHOH, ICAM1, TNFAIP3) did correlate with their findings.

It is well established that both TNF-α and GM-CSF delay neutrophil apoptosis. However, our data reveals that the genes regulating apoptosis are differentially expressed during stimulation with these two cytokines. Analysis of the expression levels of 58 apoptosis-related genes predicted differential activation of two transcription factor families. NF-κB was predicted to be activated by TNF-α, whereas STAT was predicted to be activated by GM-CSF. This prediction was validated using chemical inhibitors of both transcription factors in functional assays on healthy neutrophils incubated with both stimuli. We were able to confirm activation of NF-κB by TNF-α (and not GM-CSF), and STAT3 by GM-CSF (and not TNF-α) by Western blotting. In addition, the anti-apoptotic effects of TNF-α and GM-CSF on neutrophils were abrogated by inhibitors of NF-κB and JAK/STAT, respectively. TNF-α has previously been shown to activate the NF-κB transcription factor in neutrophils via the rapid degradation of IκB-α [51], [52] We have previously observed that NF-κB is activated in peripheral blood neutrophils from patients with RA [14], a disease that is characterised by increased levels of TNF-α and decreased levels of neutrophil apoptosis. Interestingly, RA patients who successfully responded to TNF-α therapy showed significantly less NF-κB activation in their neutrophils post-therapy compared to pre-therapy levels [14]. GM-CSF, on the other hand, cannot directly activate members of the NF-κB family. However, when GM-CSF stimulated neutrophils are allowed to adhere to surfaces such as fibronectin, a co-stimulatory signal via β_2_-integrin (CD11b/CD18) ligand binding can activate NF-κB [20]. As the neutrophils in our study were incubated in suspension, NF-κB would not be activated in our GM-CSF dataset. IPA predicted that STAT transcription family members were activated by GM-CSF, and indeed incubation of healthy neutrophils with GM-CSF in the presence of a JAK/STAT inhibitor completely abrogated the delay in apoptosis seen in GM-CSF only treated neutrophils. GM-CSF has previously been reported to activate STAT3 and STAT5 in neutrophils [53]. However, its effect on neutrophil apoptosis has, until now, been attributed to increasing stability of proteins such as Mcl-1 [15] and through the delay in activation of caspases [53].

The most up-regulated genes in TNF-α primed neutrophils shown in Table 1 included inhibitors of NF-κB signalling (NFKBIA, NFKBIE, TNFAIP3), and in GM-CSF primed neutrophils included inhibitors of STAT signalling (CISH, SOCS3). This would suggest that priming neutrophils with these cytokines, not only activates NF-κB or STAT signalling, but additionally induces expression of inhibitors of these signalling pathways [52], [54], [55]. This mechanism can thus lead to the fine tuning of gene expression during an inflammatory response.

In conclusion, we demonstrate here the first study of the neutrophil transcriptome analysed by RNA-seq with and without priming *in vitro* with two cytokines, TNF-α and GM-CSF, which are commonly elevated during *in vivo* inflammation. We show that the rapid change in phenotype associated with priming is largely independent of priming agent, as it does not rely on *de novo* protein expression. However, priming also initiated activation of transcription factors specific to the two priming agents, which resulted in the differential expression of >500 genes controlling the post-priming phenotype of the neutrophil. These dramatic transcriptomic changes are likely to have important consequences during *in vivo* inflammation, in particular in determining how differently primed neutrophils respond to secondary agonists at sites of inflammation, and how neutrophil activation is modulated by anti-inflammatory therapies. We demonstrate that whole transcriptome analysis can be applied to quantify changes in transcript levels following neutrophil stimulation *in vitro* and we suggest that this approach can also be successfully used to measure changes in the neutrophil transcriptome during inflammation or inflammatory disease, and that these expression profiles can be used to predict neutrophil phenotype in disease.

## Supporting Information

Table S1 **Summary of sequencing read alignments**. Illumina reads were mapped using default TopHat parameters, reporting only uniquely mapped reads. SOLiD reads were mapped using a modified protocol using Bowtie and TopHat as stated in Methods S1.
(DOCX)

Table S2 **Primer sequences used in real-time PCR.**
(DOCX)

Table S3 **Gene Ontology analysis of the genes significantly DE between GM-CSF treated neutrophils compared to TNF-α treated neutrophils.** GO analysis was carried out using DAVID, and revealed significant enrichment of a number of GO categories relating to apoptosis (shown in bold) (FDR adjusted q-value ≤0.05).
(DOCX)

Methods S1 **Supplementary methods.**
(DOCX)

## References

[1] Wright HL, Moots RJ, Bucknall RC, Edwards SW (2010) Neutrophil function in inflammation and inflammatory diseases. Rheumatology 49: 1618–1631. 20338884 10.1093/rheumatology/keq045

[2] Midgley A, McLaren Z, Moots RJ, Edwards SW, Beresford MW (2009) The role of neutrophil apoptosis in juvenile-onset systemic lupus erythematosus. Arthritis Rheum 60: 2390–2401. 19644848 10.1002/art.24634

[3] Naylor EJ, Bakstad D, Biffen M, Thong B, Calverley P, et al (2007) Haemophilus influenzae induces neutrophil necrosis: a role in chronic obstructive pulmonary disease? Am J Respir Cell Mol Biol 37: 135–143. 17363778 10.1165/rcmb.2006-0375OC

[4] Fossati G, Moots RJ, Bucknall RC, Edwards SW (2002) Differential role of neutrophil Fcgamma receptor IIIB (CD16) in phagocytosis, bacterial killing, and responses to immune complexes. Arthritis Rheum 46: 1351–1361. 12115243 10.1002/art.10230

[5] Mantovani A, Cassatella MA, Costantini C, Jaillon S (2011) Neutrophils in the activation and regulation of innate and adaptive immunity. Nat Rev Immunol 11: 519–531. 21785456 10.1038/nri3024

[6] Momohara S, Kashiwazaki S, Inoue K, Saito S, Nakagawa T (1997) Elastase from polymorphonuclear leukocyte in articular cartilage and synovial fluids of patients with rheumatoid arthritis. Clin Rheumatol 16: 133–140. 9093794 10.1007/BF02247841

[7] Wong SH, Francis N, Chahal H, Raza K, Salmon M, et al (2009) Lactoferrin is a survival factor for neutrophils in rheumatoid synovial fluid. Rheumatology 48: 39–44. 19029133 10.1093/rheumatology/ken412 PMC2639483

[8] Sopata I, Wize J, Filipowicz-Sosnowska A, Stanislawska-Biernat E, Brzezinska B, et al (1995) Neutrophil gelatinase levels in plasma and synovial fluid of patients with rheumatic diseases. Rheumatol Int 15: 9–14. 7652465 10.1007/BF00286763

[9] Wright HL, Bucknall RC, Moots RJ, Edwards SW (2012) Analysis of SF and plasma cytokines provides insights into the mechanisms of inflammatory arthritis and may predict response to therapy. Rheumatology 51: 451–459. 22179732 10.1093/rheumatology/ker338

[10] Vlahos R, Wark PA, Anderson GP, Bozinovski S (2012) Glucocorticosteroids differentially regulate MMP-9 and neutrophil elastase in COPD. PLoS One 7: e33277. 22413009 10.1371/journal.pone.0033277 PMC3296684

[11] Wipke BT, Allen PM (2001) Essential role of neutrophils in the initiation and progression of a murine model of rheumatoid arthritis. J Immunol 167: 1601–1608. 11466382 10.4049/jimmunol.167.3.1601

[12] Taylor PC, Peters AM, Paleolog E, Chapman PT, Elliott MJ, et al (2000) Reduction of chemokine levels and leukocyte traffic to joints by tumor necrosis factor alpha blockade in patients with rheumatoid arthritis. Arthritis Rheum 43: 38–47. 10643698 10.1002/1529-0131(200001)43:1<38::AID-ANR6>3.0.CO;2-L

[13] den Broeder AA, Wanten GJ, Oyen WJ, Naber T, van Riel PL, et al (2003) Neutrophil migration and production of reactive oxygen species during treatment with a fully human anti-tumor necrosis factor-alpha monoclonal antibody in patients with rheumatoid arthritis. J Rheumatol 30: 232–237. 12563673

[14] Wright HL, Chikura B, Bucknall RC, Moots RJ, Edwards SW (2011) Changes in expression of membrane TNF, NF-kappaB activation and neutrophil apoptosis during active and resolved inflammation. Ann Rheum Dis 70: 537–543. 21109521 10.1136/ard.2010.138065

[15] Derouet M, Thomas L, Cross A, Moots RJ, Edwards SW (2004) Granulocyte macrophage colony-stimulating factor signaling and proteasome inhibition delay neutrophil apoptosis by increasing the stability of Mcl-1. J Biol Chem 279: 26915–26921. 15078892 10.1074/jbc.M313875200

[16] Cross A, Moots RJ, Edwards SW (2008) The dual effects of TNFalpha on neutrophil apoptosis are mediated via differential effects on expression of Mcl-1 and Bfl-1. Blood 111: 878–884. 17942758 10.1182/blood-2007-05-087833

[17] Dewas C, Dang PM, Gougerot-Pocidalo MA, El-Benna J (2003) TNF-alpha induces phosphorylation of p47(phox) in human neutrophils: partial phosphorylation of p47phox is a common event of priming of human neutrophils by TNF-alpha and granulocyte-macrophage colony-stimulating factor. J Immunol 171: 4392–4398. 14530365 10.4049/jimmunol.171.8.4392

[18] Moulding DA, Hart CA, Edwards SW (1999) Regulation of neutrophil FcgammaRIIIb (CD16) surface expression following delayed apoptosis in response to GM-CSF and sodium butyrate. J Leukoc Biol 65: 875–882. 10380913 10.1002/jlb.65.6.875

[19] Belostocki K, Park MS, Redecha PB, Masuda E, Salmon JE, et al (2005) FcgammaRIIa is a target for modulation by TNFalpha in human neutrophils. Clin Immunol 117: 78–86. 16084773 10.1016/j.clim.2005.07.001

[20] Kettritz R, Choi M, Rolle S, Wellner M, Luft FC (2004) Integrins and cytokines activate nuclear transcription factor-kappaB in human neutrophils. J Biol Chem 279: 2657–2665. 14613935 10.1074/jbc.M309778200

[21] Trapnell C, Pachter L, Salzberg SL (2009) TopHat: discovering splice junctions with RNA-Seq. Bioinformatics 25: 1105–1111. 19289445 10.1093/bioinformatics/btp120 PMC2672628

[22] Trapnell C, Roberts A, Goff L, Pertea G, Kim D, et al (2012) Differential gene and transcript expression analysis of RNA-seq experiments with TopHat and Cufflinks. Nat Protoc 7: 562–578. 22383036 10.1038/nprot.2012.016 PMC3334321

[23] Langmead B, Trapnell C, Pop M, Salzberg SL (2009) Ultrafast and memory-efficient alignment of short DNA sequences to the human genome. Genome Biol 10: R25. 19261174 10.1186/gb-2009-10-3-r25 PMC2690996

[24] Trapnell C, Williams BA, Pertea G, Mortazavi A, Kwan G, et al (2010) Transcript assembly and quantification by RNA-Seq reveals unannotated transcripts and isoform switching during cell differentiation. Nat Biotechnol 28: 511–515. 20436464 10.1038/nbt.1621 PMC3146043

[25] Ramskold D, Wang ET, Burge CB, Sandberg R (2009) An abundance of ubiquitously expressed genes revealed by tissue transcriptome sequence data. PLoS Comput Biol 5: e1000598. 20011106 10.1371/journal.pcbi.1000598 PMC2781110

[26] Rowley JW, Oler AJ, Tolley ND, Hunter BN, Low EN, et al (2011) Genome-wide RNA-seq analysis of human and mouse platelet transcriptomes. Blood 118: e101–111. 21596849 10.1182/blood-2011-03-339705 PMC3193274

[27] Saeed AI, Bhagabati NK, Braisted JC, Liang W, Sharov V, et al (2006) TM4 microarray software suite. Methods Enzymol 411: 134–193. 16939790 10.1016/S0076-6879(06)11009-5

[28] Huang da W, Sherman BT, Lempicki RA (2009) Systematic and integrative analysis of large gene lists using DAVID bioinformatics resources. Nat Protoc 4: 44–57. 19131956 10.1038/nprot.2008.211

[29] Pfaffl MW (2001) A new mathematical model for relative quantification in real-time RT-PCR. Nucleic Acids Research 29: 2002–2007. 10.1093/nar/29.9.e45 PMC55695 11328886

[30] Edwards SW (1996) The O-2 Generating NADPH Oxidase of Phagocytes: Structure and Methods of Detection. Methods 9: 563–577. 8812712 10.1006/meth.1996.0064

[31] Dransfield I, Buckle AM, Savill JS, McDowall A, Haslett C, et al (1994) Neutrophil apoptosis is associated with a reduction in CD16 (Fc gamma RIII) expression. J Immunol 153: 1254–1263. 8027553

[32] Mortazavi A, Williams BA, McCue K, Schaeffer L, Wold B (2008) Mapping and quantifying mammalian transcriptomes by RNA-Seq. Nat Methods 5: 621–628. 18516045 10.1038/nmeth.1226 PMC13303166

[33] Wilhelm BT, Briau M, Austin P, Faubert A, Boucher G, et al (2011) RNA-seq analysis of 2 closely related leukemia clones that differ in their self-renewal capacity. Blood 117: e27–38. 20980679 10.1182/blood-2010-07-293332

[34] Huang W, Nadeem A, Zhang B, Babar M, Soller M, et al (2012) Characterization and comparison of the leukocyte transcriptomes of three cattle breeds. PLoS One 7: e30244. 22291923 10.1371/journal.pone.0030244 PMC3264571

[35] Kobayashi SD, Voyich JM, Buhl CL, Stahl RM, DeLeo FR (2002) Global changes in gene expression by human polymorphonuclear leukocytes during receptor-mediated phagocytosis: cell fate is regulated at the level of gene expression. Proc Natl Acad Sci U S A 99: 6901–6906. 11983860 10.1073/pnas.092148299 PMC124501

[36] Kobayashi SD, Voyich JM, Whitney AR, DeLeo FR (2005) Spontaneous neutrophil apoptosis and regulation of cell survival by granulocyte macrophage-colony stimulating factor. J Leukoc Biol 78: 1408–1418. 16204629 10.1189/jlb.0605289

[37] Ashburner M, Ball CA, Blake JA, Botstein D, Butler H, et al (2000) Gene ontology: tool for the unification of biology. The Gene Ontology Consortium. Nat Genet 25: 25–29. 10802651 10.1038/75556 PMC3037419

[38] Fessler MB, Malcolm KC, Duncan MW, Worthen GS (2002) A genomic and proteomic analysis of activation of the human neutrophil by lipopolysaccharide and its mediation by p38 mitogen-activated protein kinase. J Biol Chem 277: 31291–31302. 11943771 10.1074/jbc.M200755200

[39] Zhang X, Kluger Y, Nakayama Y, Poddar R, Whitney C, et al (2004) Gene expression in mature neutrophils: early responses to inflammatory stimuli. J Leukoc Biol 75: 358–372. 14634056 10.1189/jlb.0903412

[40] de Kleijn S, Kox M, Sama IE, Pillay J, van Diepen A, et al (2012) Transcriptome kinetics of circulating neutrophils during human experimental endotoxemia. PLoS One 7: e38255. 22679495 10.1371/journal.pone.0038255 PMC3367952

[41] Malone JH, Oliver B (2011) Microarrays, deep sequencing and the true measure of the transcriptome. BMC Biol 9: 34. 21627854 10.1186/1741-7007-9-34 PMC3104486

[42] Marioni JC, Mason CE, Mane SM, Stephens M, Gilad Y (2008) RNA-seq: an assessment of technical reproducibility and comparison with gene expression arrays. Genome Res 18: 1509–1517. 18550803 10.1101/gr.079558.108 PMC2527709

[43] Fu X, Fu N, Guo S, Yan Z, Xu Y, et al (2009) Estimating accuracy of RNA-Seq and microarrays with proteomics. BMC Genomics 10: 161. 19371429 10.1186/1471-2164-10-161 PMC2676304

[44] Su Z, Li Z, Chen T, Li QZ, Fang H, et al (2011) Comparing next-generation sequencing and microarray technologies in a toxicological study of the effects of aristolochic acid on rat kidneys. Chem Res Toxicol 24: 1486–1493. 21834575 10.1021/tx200103b

[45] Jog NR, Rane MJ, Lominadze G, Luerman GC, Ward RA, et al (2007) The actin cytoskeleton regulates exocytosis of all neutrophil granule subsets. Am J Physiol Cell Physiol 292: C1690–1700. 17202227 10.1152/ajpcell.00384.2006

[46] Cassatella MA (1999) Neutrophil-derived proteins: selling cytokines by the pound. Adv Immunol 73: 369–509. 10399011 10.1016/s0065-2776(08)60791-9

[47] Iwamoto T, Okamoto H, Toyama Y, Momohara S (2008) Molecular aspects of rheumatoid arthritis: chemokines in the joints of patients. FEBS J 275: 4448–4455. 18662305 10.1111/j.1742-4658.2008.06580.x

[48] Szekanecz Z, Kim J, Koch AE (2003) Chemokines and chemokine receptors in rheumatoid arthritis. Semin Immunol 15: 15–21. 12495637 10.1016/s1044-5323(02)00124-0

[49] Hatano Y, Kasama T, Iwabuchi H, Hanaoka R, Takeuchi HT, et al (1999) Macrophage inflammatory protein 1 alpha expression by synovial fluid neutrophils in rheumatoid arthritis. Ann Rheum Dis 58: 297–302. 10225815 10.1136/ard.58.5.297 PMC1752877

[50] Beaulieu AD, McColl SR (1994) Differential expression of two major cytokines produced by neutrophils, interleukin-8 and the interleukin-1 receptor antagonist, in neutrophils isolated from the synovial fluid and peripheral blood of patients with rheumatoid arthritis. Arthritis Rheum 37: 855–859. 8003057 10.1002/art.1780370613

[51] Ward C, Chilvers ER, Lawson MF, Pryde JG, Fujihara S, et al (1999) NF-kappaB activation is a critical regulator of human granulocyte apoptosis in vitro. J Biol Chem 274: 4309–4318. 9933632 10.1074/jbc.274.7.4309

[52] McDonald PP, Bald A, Cassatella MA (1997) Activation of the NF-kappaB pathway by inflammatory stimuli in human neutrophils. Blood 89: 3421–3433. 9129050

[53] Fortin CF, Larbi A, Dupuis G, Lesur O, Fulop T (2007) GM-CSF activates the Jak/STAT pathway to rescue polymorphonuclear neutrophils from spontaneous apoptosis in young but not elderly individuals. Biogerontology 8: 173–187. 17086367 10.1007/s10522-006-9067-1

[54] Nelson DE, Ihekwaba AE, Elliott M, Johnson JR, Gibney CA, et al (2004) Oscillations in NF-kappaB signaling control the dynamics of gene expression. Science 306: 704–708. 15499023 10.1126/science.1099962

[55] Krebs DL, Hilton DJ (2000) SOCS: physiological suppressors of cytokine signaling. J Cell Sci 113 (Pt 16): 2813–2819. 10.1242/jcs.113.16.2813 10910765

