# Supplementary material for: RNA-Seq Reveals Activation of Both Common and Cytokine-Specific Pathways following Neutrophil Priming
Source: PLoS One. 2013 Mar 6;8(3):e58598. doi: 10.1371/journal.pone.0058598 (PMC3590155; doi:10.1371/journal.pone.0058598)
Supplement: Table S1 — Summary of sequencing read alignments. Illumina reads were mapped using default TopHat parameters, reporting only uniquely mapped reads. SOLiD reads were mapped using a modified protocol using Bowtie and TopHat as stated in Methods S1. (DOCX) [file pone.0058598.s001.docx]

**Table S1**

|  |  | **Untreated** | **GM-CSF** | **TNF-α** |
| --- | --- | --- | --- | --- |
| **SOLiD** | Library size | 127,885,988 | 75,544,747 | 69,005,645 |
|  | Total Mapped Reads | 49,354,278 | 25,752,506 | 23,241,076 |
| **Illumina-1** | Library size | 66,552,453 | 64,445,900 | 65,625,666 |
|  | Total Mapped Reads | 58,509,014 | 56,916,427 | 57,963,723 |
| **Illumina-2** | Library size | 49,771,919 | 46,782,183 | 46,388,772 |
|  | Total Mapped Reads | 44,531,046 | 41,988,614 | 41,521,993 |
