# Supplementary material for: RNA-Seq Reveals Activation of Both Common and Cytokine-Specific Pathways following Neutrophil Priming
Source: PLoS One. 2013 Mar 6;8(3):e58598. doi: 10.1371/journal.pone.0058598 (PMC3590155; doi:10.1371/journal.pone.0058598)
Supplement: Table S2 — Primer sequences used in real-time PCR. (DOCX) [file pone.0058598.s002.docx]

**Table S2**

| **Gene of Interest** | **Primer Sequence (5’ to 3’)** | **Product size (bp)** |
| --- | --- | --- |
| **FADD-f** | CACAGACCACCTGCTTCTGA | 176 |
| **FADD-r** | CTGGACACGGTTCCAACTTT |  |
| **FOS-f** | CTCCGGTGGTCACCTGTACT | 137 |
| **FOS-r** | GTCAGAGGAAGGCTCATTGC |  |
| **ICAM1-f** | AGCTTCGTGTCCTGTATGGCCC | 128 |
| **ICAM1-r** | ACACTTGAGCTCGGGCAATGGG |  |
| **IL1B-f** | CACTACAGCAAGGGCTTCAGGC | 98 |
| **IL1B-r** | TTCTCCTGGAAGGTCTGTGGGC |  |
| **IL8-f** | AAAAGCCACCGGAGCACTCCAT | 143 |
| **IL8-r** | AGAGCCACGGCCAGCTTGGA |  |
| **JUN-f** | TGGCAGAGTCCCGGAGCGAA | 121 |
| **JUN-r** | CGAAGCTGAGCGCACGTCCT |  |
| **NAMPT-f** | GCCAGCAGGGAATTTTGTTA | 100 |
| **NAMPT-r** | TGTCACCTTGCCATTCTTGA |  |
| **SOCS3-f** | CTGGTCCCCTCCCGGTTGGT | 112 |
| **SOCS3-r** | TGTTGGCGGCCGTGAAGTCC |  |
| **TNF-f** | CAGAGGGCCTGTACCTCATC | 219 |
| **TNF-r** | GGAAGACCCCTCCCAGATAG |  |
| **ACTB-f** | CATCGAGCACGGCATCGTCA | 211 |
| **ACTB-r** | TAGCACAGCCTGGACAGCAAC |  |
| **B2M-f** | ACTGAATTCACCCCCACTGA | 114 |
| **B2M-r** | CCTCCATGATGCTGCTTACA |  |
| **GAPDH-f** | CTCAACGACCACTTTGTCAAGCTCA | 106 |
| **GAPDH-r** | GGTCTTACTCCTTGGAGGCCATGTG |  |
| **PPIA-f** | GCTTTGGGTCCAGGAATGG | 60 |
| **PPIA-r** | GTTGTCCACAGTCAGCCATGGT |  |
