# Supplementary material for: RNA-Seq Reveals Activation of Both Common and Cytokine-Specific Pathways following Neutrophil Priming
Source: PLoS One. 2013 Mar 6;8(3):e58598. doi: 10.1371/journal.pone.0058598 (PMC3590155; doi:10.1371/journal.pone.0058598)
Supplement: Table S3 — Gene Ontology analysis of the genes significantly DE between GM-CSF treated neutrophils compared to TNF-α treated neutrophils. GO analysis was carried out using DAVID, and revealed significant enrichment of a number of GO categories relating to apoptosis (shown in bold) (FDR adjusted q-value ≤0.05). (DOCX) [file pone.0058598.s003.docx]

**Table S3**

| **GO Term** | **GO Category** | **No of Genes** | **FDR (q-value)** |
| --- | --- | --- | --- |
| **GO:0042981** | **regulation of apoptosis** | **58** | **3.17E-06** |
| **GO:0043067** | **regulation of programmed cell death** | **58** | **4.61E-06** |
| **GO:0010941** | **regulation of cell death** | **58** | **5.21E-06** |
| GO:0006952 | defense response | 52 | 2.81E-07 |
| GO:0006955 | immune response | 52 | 1.51E-05 |
| **GO:0016265** | **death** | **51** | **1.84E-04** |
| GO:0010604 | positive regulation of macromolecule metabolic process | 51 | 2.49E-02 |
| GO:0009611 | response to wounding | 50 | 1.43E-08 |
| **GO:0008219** | **cell death** | **50** | **3.72E-04** |
| GO:0031328 | positive regulation of cellular biosynthetic process | 49 | 2.23E-04 |
| GO:0009891 | positive regulation of biosynthetic process | 49 | 3.48E-04 |
| **GO:0012501** | **programmed cell death** | **48** | **1.78E-05** |
| **GO:0006915** | **apoptosis** | **47** | **3.16E-05** |
| GO:0051173 | positive regulation of nitrogen compound metabolic process | 46 | 6.20E-04 |
| GO:0010557 | positive regulation of macromolecule biosynthetic process | 46 | 9.51E-04 |
| GO:0006357 | regulation of transcription from RNA polymerase II promoter | 46 | 1.58E-02 |
| GO:0045935 | positive regulation of nucleobase, nucleoside, nucleotide and nucleic acid metabolic process | 42 | 9.88E-03 |
| GO:0010628 | positive regulation of gene expression | 41 | 4.15E-03 |
| GO:0045941 | positive regulation of transcription | 40 | 5.06E-03 |
| GO:0006954 | inflammatory response | 39 | 3.60E-09 |
| GO:0019220 | regulation of phosphate metabolic process | 36 | 6.54E-03 |
| GO:0051174 | regulation of phosphorus metabolic process | 36 | 6.54E-03 |
| GO:0007243 | protein kinase cascade | 35 | 3.18E-05 |
| GO:0042325 | regulation of phosphorylation | 35 | 7.09E-03 |
| GO:0045893 | positive regulation of transcription, DNA-dependent | 34 | 2.99E-02 |
| GO:0051254 | positive regulation of RNA metabolic process | 34 | 3.63E-02 |
| GO:0043066 | negative regulation of apoptosis | 32 | 4.10E-04 |
| GO:0043069 | negative regulation of programmed cell death | 32 | 5.58E-04 |
| **GO:0060548** | **negative regulation of cell death** | **32** | **5.94E-04** |
| GO:0001775 | cell activation | 30 | 4.67E-05 |
| GO:0045321 | leukocyte activation | 29 | 4.09E-06 |
| GO:0001817 | regulation of cytokine production | 24 | 1.92E-05 |
| GO:0046649 | lymphocyte activation | 23 | 4.93E-04 |
| **GO:0006916** | **anti-apoptosis** | **22** | **3.49E-03** |
| GO:0001819 | positive regulation of cytokine production | 15 | 1.26E-03 |
| GO:0050867 | positive regulation of cell activation | 15 | 1.62E-02 |
| GO:0032496 | response to lipopolysaccharide | 14 | 1.20E-03 |
| GO:0002237 | response to molecule of bacterial origin | 14 | 4.41E-03 |
| GO:0002696 | positive regulation of leukocyte activation | 14 | 4.57E-02 |
| GO:0031349 | positive regulation of defense response | 12 | 2.70E-02 |
| GO:0051100 | negative regulation of binding | 11 | 2.27E-02 |
| GO:0043433 | negative regulation of transcription factor activity | 10 | 1.52E-02 |
| GO:0043392 | negative regulation of DNA binding | 10 | 4.41E-02 |
| GO:0032675 | regulation of interleukin-6 production | 9 | 2.13E-02 |
