# Supplementary material for: RNA-Seq Reveals Activation of Both Common and Cytokine-Specific Pathways following Neutrophil Priming
Source: PLoS One. 2013 Mar 6;8(3):e58598. doi: 10.1371/journal.pone.0058598 (PMC3590155; doi:10.1371/journal.pone.0058598)
Supplement: Methods S1 — Supplementary methods. (DOCX) [file pone.0058598.s004.docx]

**Methods S1**

**Read Mapping.** Reads were mapped to the human genome (hg19) using TopHat (v1.4.1). For Illumina data, default read mapping parameters were used with the addition of the -g / --max-multi-hits option set as 1, to report only uniquely mapping reads. For SOLiD data, preliminary analysis of read quality scores showed a decrease in quality of SOLiD reads towards the 3’ end of the read (data not shown), and consequently reads were trimmed by 10 bases from the 3’ end and first mapped to the human genome using Bowtie (v0.12.7). Reads not uniquely mapped by Bowtie were then mapped using TopHat and the two datasets merged. The number of reads mapped in each library is shown in Supplementary Table 1.

**Gene Annotation**. Gene annotation and calculation of RPKM values was carried out using Cufflinks (v1.3.0) with the provision of a GTF annotation file (hg19).

**Statistical Analysis**. Differential expression (DE) analysis was carried out using the Cufflinks tool Cuffdiff, applying a false discovery rate (FDR) of 5%. Cuffdiff analysis was visualised using the MeV. All other statistical analysis was carried out in R using Student’s t-test or Pearson Correlation test, as stated.

**Bioinformatics.** Gene ontology analysis of genes with significant DE (5% FDR) between untreated and cytokine treated samples was carried out using DAVID. The functional analyses were generated through the use of IPA (Ingenuity^®^ Systems, [www.ingenuity.com)](http://www.ingenuity.com)). Analysis identified the pathways from the IPA library of canonical pathways that were most significant to the data set. Molecules from the data set that met the 1.5-fold change (cytokine treated vs untreated cells) cut-off and were associated with a canonical pathway in the Ingenuity Knowledge Base were considered for the analysis. The significance of the association between the data set and the canonical pathway was measured in 2 ways: 1) a ratio of the number of molecules from the data set that mapped to the pathway, divided by the total number of molecules that mapped to the canonical pathway. 2) Fisher’s exact test was used to calculate a p‐value, determining the probability that the association between the genes in the dataset and the canonical pathway was explained by chance alone.
